# The obese gut microbiome across the epidemiologic transition

**DOI:** 10.1186/s12982-015-0044-5

**Published:** 2016-01-11

**Authors:** Lara R. Dugas, Miles Fuller, Jack Gilbert, Brian T. Layden

**Affiliations:** Public Health Sciences, Stritch School of Medicine, Loyola University Chicago, 2160 S. 1st Ave, Maywood, IL 60153 USA; Division of Endocrinology, Metabolism and Molecular Medicine, Northwestern University, Evanston, USA; Argonne National Laboratory, Biosciences Department, Institute for Genomic and Systems Biology, 9700 South Cass Avenue, Argonne, IL 60439 USA; Department of Ecology and Evolution, University of Chicago, 1101 E 57th Street, Chicago, IL 60637 USA; Marine Biological Laboratory, 7 MBL Street, Woods Hole, MA 02543 USA; College of Environmental and Resource Sciences, Zhejiang University, Hangzhou, 310058 China; Jesse Brown Veterans Affairs Medical Center, Chicago, IL USA

**Keywords:** Obesity, Gut microbiome, Geographical differences

## Abstract

The obesity epidemic has emerged over the past few decades and is thought to be a result of both genetic and environmental factors. A newly identified factor, the gut microbiota, which is a bacterial ecosystem residing within the gastrointestinal tract of humans, has now been implicated in the obesity epidemic. Importantly, this bacterial community is impacted by external environmental factors through a variety of undefined mechanisms. We focus this review on how the external environment may impact the gut microbiota by considering, the host’s geographic location ‘human geography’, and behavioral factors (diet and physical activity). Moreover, we explore the relationship between the gut microbiota and obesity with these external factors. And finally, we highlight here how an epidemiologic model can be utilized to elucidate causal relationships between the gut microbiota and external environment independently and collectively, and how this will help further define this important new factor in the obesity epidemic.

## Background

In 2014, the World Health Organization (WHO) estimated that approximately half a billion adults were obese, a doubling in the prevalence since 1980 [1]. Considering this statistic, the obesity epidemic is a global health issue, impacting both the industrialized and developing world [2, 3], where the societal impact is felt through the multitude of comorbidities occurring with obesity including type 2 diabetes [4, 5], nonalcoholic fatty liver disease [6] and cardiovascular diseases [7, 8]. These medical complications and the international socioeconomic implications are considerable motivations for investigating the epidemic. The goal of this review is to provide a novel epidemiologic perspective of viewing and studying the global obesity epidemic, in light of the newly discovered factor, the gut microbiota.

Obesity is defined as excess adiposity where an imbalance in two processes; caloric intake and physical activity levels, are thought to be the key drivers [9, 10]. Genetic and environmental factors impact both these two processes through complex mechanisms [11]. Genome wide association studies have been helping us untangle this complex genetic landscape for over a decade [12]; however, genetic polymorphisms alone do not explain the obesity epidemic. Many environmental factors contribute to the obesity epidemic, where these environmental factors are related and definable largely by the geographical location of the individual, as the location defines many influences of the obesity epidemic (such as diet and physical activity). As compared to our genetics which could not have changed dramatically over the time period that the obesity epidemic has emerged, many key environmental factors have been altered over this timeframe, most notably shifts in diet and physical activity habits and patterns [3, 13], and others factors including the rise of the built environment [14], social and economic factors [15], environmental endocrine disruptors [16], and co-morbid medical conditions [17]. Interestingly, some of these environmental factors interact with our genes through epigenetic mechanisms [18]. One example, as reported by Rosenquist et al. [19], where significant gene-by-birth cohort interactions with the FTO variant (rs993609) occurred, was observed by using longitudinal data from the Framingham study, were the genetic impact of this variant on BMI over time, indicates the influence of the changing environmental factors. Taken together, genetic and environmental factors are driving the obesity epidemic through complex interactions.

Recently, a new environmental factor, the gut microbiota (defined as microbes that live in the gastrointestinal tract) has been implicated as a factor in the obese phenotype [20–27]. This novel factor is an ecosystem in itself, comprised of 500–1000 species per person, and the sum genetic potential of these diverse assemblages can exceed 100 fold the number of human genes [28, 29]. This results in many of these bacterial genes having unique functions that complement the genetic repertoire of humans [30]. Of importance, until recently scientists have found it challenging to study the gut microbiota, as many of these organisms cannot be grown in culture, thus limiting our ability to investigate their individual physiological and metabolic potential. The advent of new genetic sequencing approaches has enabled us to describe the diversity and functional potential of these assemblages. Metagenomic approaches, particularly, enable the functional potential of the assemblage to be linked to its phylogenetic composition. Because of the advances in sequencing approaches, the gut microbiota is now approachable to be studied [31, 32].

As the gut microbiota appears to be influenced by many external factors in the host’s environment [33], investigation of this microbial ecosystem in relation to other influencing external factors is needed [34, 35]. Thus, studying this internal ecosystem independently and relative to external factors in the obesity epidemic is needed, and will help us understand the nature of the obesity epidemic and the novel role of the gut microbiome. As the geographical location of the host defines many of these external factors that influence the gut microbiota, we systematically explore this topic (1) by providing background on how human geography is related to the obesity epidemic (‘Human Geography and Obesity’), (2) assessing the literature on the gut microbiota relative to one’s geography (‘Gut Microbiota and Geography’) (3) describing the key studies relating the gut microbiota to obesity (‘Gut Microbiota and Obesity’) and finally, (4) other key related factors in this interaction with the obese gut microbiota (‘Human Geography, Diet and Physical Activity, and the Obesity-Associated Microbiota’). Moreover, this review provides a rationale for employing a global epidemiologic model for studying the associations between the gut microbiota and the development of obesity, which allows capturing geographical diverse external environmental factors.

## Human geography and obesity

Human geography, as defined by a particular group of individuals in a location, has unique relationship to obesity. In large part, this interaction of human geography with obesity is related to the development status of the people within this geographic location. For example, the burden of the obesity epidemic is felt most in developed countries [36, 37], and is related to the human development index (HDI) of the country. This index is a United Nations Index describing the level of development of that population/country [38].

Exemplifying this, one of the first large multi-country cohort studies, initiated in the early 1990’s, titled “The International Collaborative Study on Hypertension in Blacks” [39, 40], recruited over 9000 adults living in seven countries, including the US (urban Chicago), Africa (rural and urban Nigeria and Cameroon), and the Caribbean (Jamaica, Barbados, and St. Lucia), and found the prevalence of obesity ranged from approximately 1 % in African cohorts to 36 % in the US [41, 42]. More recently (2010–2013), we conducted a large multi-country study following a cohort of 2500 young adults of African descent from five countries in the Modeling the Epidemiologic Transition Study (METS). Participants were from the US (urban), Ghana (rural), South Africa (peri-urban), Jamaica (urban) and the Seychelles (urban) [2]. We found that the prevalence of obesity in adults of African origin continues to mostly present in a continuum reflecting each countries HDI ranking, i.e. lowest in Ghana and highest in the US. In METS, the prevalence of obesity ranges from 1.4 % in Ghanaian men to 64 % in US women [2]. While the prevalence of obesity has increased among all of the METS research sites, it has more than doubled among US men and increased from 42 to 67 % among the women sampled from the same urban US community over a 15 year time period (unpublished data). Ezzati et al. [43], using data from over 100 countries, found that the relationship between BMI and country stage of development was best captured using a U-shaped association, and that the rate of increasing BMI change was greatest in countries moving from low to middle development, compared to countries moving from middle to high development [43]. In fact, we have demonstrated that adults residing in Jamaica, a rapidly transiting country, experienced significantly more weight gain compared to adults living in either the USA or Nigeria over a 4 year period between 1995 and 1999 supporting this U-shaped relationship [44]. Consistent with this model, we have shown that the prevalence of obesity-related chronic disease, including hypertension [42, 45] and type 2 diabetes [46–48] in blacks of the African diaspora occurs in manner related to the resident countries HDI. Consequently, studying different populations allows us to identify these populations with changing rates of obesity and related co-morbidities. Taken together, these data indicate the country-level stage of economic development has a strong association with the population-level prevalence of obesity.

## Gut microbiota and geography

**Fig. 1:**
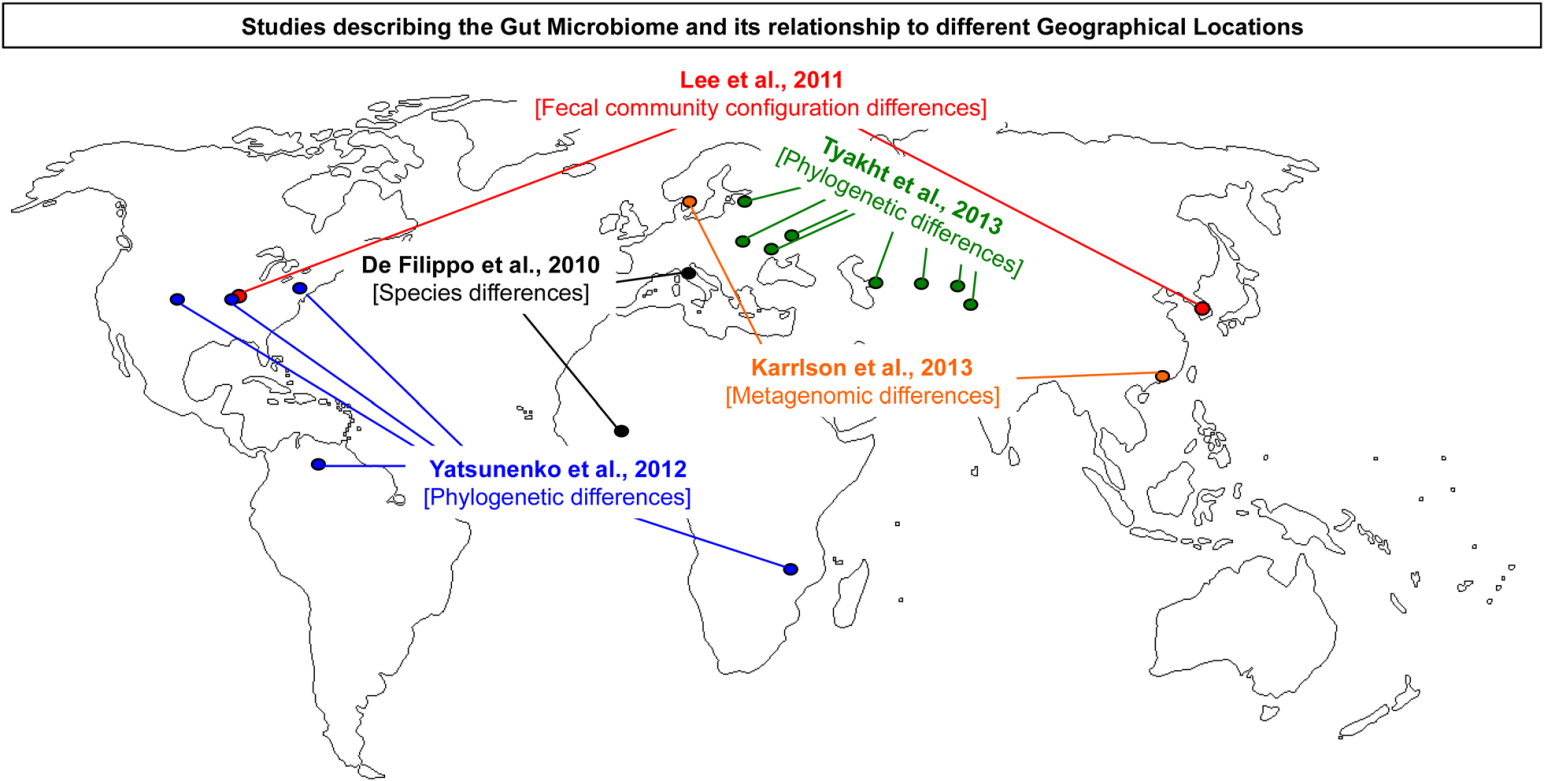
Studies describing the gut microbiome and their relationship to geographical locations

Some studies have begun to investigate the similarities and differences in the gut microbiota across populations [29, 49, 50]. In one of the first studies to examine this, Karlsson et al. [29] attempted to identify if a common gut microbiome exists across populations, where in this study, they examined 782 persons from four studies, spanning three different continents (Europe, USA and China). Overall, it was observed that differences in species, gene richness and diversity existed across populations, but that a common gut microbiome was shared, with approximately half a million microbial genes between studies (see Table 1A; Fig. 1). Another study, examining a range of individuals from birth to 70 years in populations of Venezuela, Malawi, and United States [49], found that the gut microbiota of each population had distinct overall phylogenetic composition. Looking at the collective microbiome, assessed through metagenomic shotgun sequencing of a subset of this group (110 of the 531 total individuals that had bacterial species determined), unique patterns of over and under-repressed genes was observed between populations [49]. Further, recent evidence has suggested that even visits of short duration to other geographic locations can influence the gut microbial assemblage; specifically a month long visit to Bangkok, Thailand, can dramatically influence the microbial composition of one participant’s microbiome [51]. Collectively these data indicate while shared features in the gut microbiota may exist across populations, large differences exists across populations.

**Table 1:** Key studies describing the gut microbiota and its relationship to different geographical locations (A), and key studies describing the gut microbiota and its relationship to obesity (B) are indicated

Author	Groups	Geographical effect
A		
De Filippo et al. [77]	Rural Africa vs. Italian children (1–6 years)	Species differences existed that conferred specific nutritional effects
Lee et al. [50]	Monozygotic and dizygotic US vs. Korean twins, either normal weight (BMI < 25 kg/m^2^) or overweight (BMI > 25 kg/m^2^)	Significant differences in configuration fecal communities between sites
Yatsunenko et al. [49]	Venezuelan (Amerindians), Malawian, US children (0–17 years) and adults (18–70 years)	Phylogenetic and microbiota enzymatic differences
Tyakht et al. [81]	Urban vs. rural Russian males and females (14–85 years)	Phylogenetic differences existed
Karrlson et al. [28]	Type 2 diabetes, normal- and impaired-glucose tolerance older European women (>70 years) vs. type 2 diabetes, normal and impaired glucose tolerance Chinese men and women (13–86 years)	Metagenomic cluster differed between two populations
David et al. [51]	Two US adult males (26, and 36 years)	Travel acutely altered phylogenetic taxa

Author	Groups	Obesity effect
B		
Backhed et al. [52]	Conventionally raised vs. germ-free and germ-free conventionalized	Conventional had significantly more body fat than germ-free, as did conventionalized, both eating less chow
Ley et al. [30]	Lean vs. obese humans	Relative proportion of Bacteroidetes is reduced in obesity
Turnbaugh et al. [21]	Ob/ob mice vs. ob/+ and +/+mice	Ob/ob mice increased rate of energy harvest from diet, transmission of ob/ob gut microbiome to +/+ resulted in significantly greater increase in body fat
Turnbaugh et al. [24]	Lean (BMI < 25 kg/m^2^) and overweight (BMI ≥ 25 kg/m^2^) or obese (BMI ≥ 30 kg/m^2^) twins	Lower proportion Bacteroidetes and higher proportion of Actinobacteria in obese vs. lean twins. No difference in Firmicutes between twins
Turbbaugh et al. [25]	Germ-free mice colonized with human gut microbiota, fed either low-fat, plant polysaccharide-rich or high fat (Western) diet	Increase in proportion of Firmicutes and decrease in proportion of Bacteroidetes in mice fed western diet. Off-spring from either germ-free or humanized mice indicated gut microbiome could be transmitted, sharing 83 % of class-level Taxa and 73 % genus level
Murphy et al. [56]	Ob/ob mice vs. wild type on high- or low-fat diet	Increase in Firmicutes in high-fat and ob/ob. Reduction in Bacteroidetes in ob/ob only
Lee et al. [50]	US vs Korean	Lower alpha-diversity in obesity, regardless of site
Ridaura et al. [22]	Germ-free mice transplanted with fecal microbiota from twins discordant for obesity (obese twin BMI ≥ 30 kg/m^2^)	Mice receiving obese fecal microbiota had significantly greater increase in adipose mass. Feces from mice with obese fecal microbiota had higher branched chain amino acids

While not exhaustive, these studies exemplify a simple observation, that microbial assemblages associated with the human intestinal tract are not homogeneous throughout the world. Additionally, certain microbial phylogenetic characteristics are likely globally shared on some level; however, specific genetic signatures selected for by local environmental characteristics undoubtedly occurs in geographically disparate environments. Moreover, within each population exist lifestyle factors such as diet, and physical activity which are likely influencing the observed differences in microbial community structure across different geographic environments. Thus, rather than geographic location itself driving microbial patterns, it is possible that geography is a proxy for these factorial influences.

## Gut microbiota and obesity

The human gut microbiome has been linked to the obesity epidemic [24, 52–55]. However, animal studies comprise the majority of causative evidence linking changes in microbial composition to the obesity phenotype (see Table 1B). In seminal rodent-based work by Backhed et al. [52], the gut microbiota was observed to regulate the host’s ability to harvest energy from food, thus showing its role in host fat storage [52]. Subsequent experiments suggest that gut microbiota are affected by adiposity (with higher ratios of Firmicutes to Bacteroidetes in ob/ob mice [23]. Further work has established the potential for a particular gut microbiota ecosystem to impart an obesity phenotype, where the microbiota of ob/ob (a genetic model of obesity) or lean mice where transferred to germ-free mice [20]. It was also observed that the colonization of germ-free mice with ob/ob mouse-associated gut microbiota resulted in greater weight gain and energy extraction than the colonization with lean mouse-associated gut microbiota [21]. Providing further direct evidence for the existence of a transmissible obesity microbiota, Ridaura et al. [22] transplanted uncultured gut microbiota collected from feces from adult female twin pairs, discordant for obesity into germ-free mice fed the same diets. Mice receiving the obesity gut microbiota experienced significantly greater increases in adiposity. It was also noted that the fecal biomass from the lean mice was significantly greater compared to the fecal biomass from the obese siblings. It should also be noted that changes in phyla are not always a result of obesity per se, but may simply be a reflection of the macronutrient composition changes [56].

In humans, the data are more variable, but overall most studies indicate an increase in the Firmicutes and a decrease in the Bacteroidetes phyla to be associated with obesity [20, 54], although not all studies have observed this [55, 57]. Yet, in one of the earliest human studies, Ley et al. [20] compared the gut microbiota of 12 obese individuals, following two different low calorie diets over the period of 1 year and found that at baseline, obesity was associated with fewer Bacteroidetes (p < 0.001). However, with subsequent weight loss, there were increases in the Bacteroidetes, concomitant with decreases in the Firmicutes phyla, and thus an increased Bacteroidetes/Firmicutes ratio, irrespective of diet assignment. Ferrer et al. [54] confirmed these findings comparing the gut microbiota in lean and obese individuals. However, as noted in a review by Bell [58], whether the human gut microbiota is causing obesity is not clear.

Some studies have attempted to address this challenging question. Vrieze et al. [59] did investigate the effect of transferring human gut microbiota from healthy lean individuals (BMI < 23 kg/m^2^) into male participants with the metabolic syndrome. Participants were either infused with the healthy gut microbiota (N = 9) or reinfused with their own gut microbiota (N = 9). Prior to infusion, the gut microbiota from the obese participants was characterized by an overabundance of Bacteroidetes, and lower microbial diversity compared to the lean healthy donors. Importantly, peripheral insulin sensitivity was measured before and repeated 6 weeks after the gut microbiota infusion and found to significantly increase in those participants receiving the healthy microbiota. While not answering the relationship of the gut microbiota to obesity, these data suggest a causal role of the gut microbiota in insulin sensitivity.

As such many postulated mechanisms of how the gut microbiota contributes to obesity through dysbiosis have been suggested [21, 60–63] including increased energy harvest by the obese microbiome or diet-induced changes in the gut microbiome leading to low levels of gut and systemic inflammation [60–62]. Examples of this later mechanism comes from Ding et al. [63], who reported that a combination of a high fat diet and gut microbiota induced intestinal inflammation and weight gain in mice. Similarly, Cani et al. [61] showed that following prebiotic administration, mice exhibited lower plasma cytokines as well as hepatic and oxidative stress markers as a result of prebiotic alterations in the gut microbiota. While these rodent studies are compelling, our basic understanding of our gut microbiota ecosystem remains incomplete, and multiple other mechanisms may be involved [58, 64–67], particularly in humans.

## Human geography, diet, and physical activity, and the obesity-associated microbiota

Very few studies have explicitly examined the interaction of human geography, microbial community structure and obesity. However, one provocative study invokes Bergmann’s rule, that humans are heavier in higher latitudes compared to lower latitudes, and suggests that this may be the result of the functional ability of the microbiome to extract energy from food [68]. The authors of this study cite the higher Firmicutes to Bacteroidetes ratio in high latitude populations as evidence for this relationship. Another study by Arumugun et al. [57] using DNA sequence homology to examine phylogenetic composition found significant variation among 39 individuals from six different countries. Analyses revealed three distinct profiles among the six different nations represented; identifiable by the variation in the levels of one of three genera: Bacteroides, Prevotella and Ruminococcus. The authors, however, could not account for any genera-derived BMI effects in these samples, but did find phylogenetic differences which suggested that the variation in the dominance of each phyla among the countries reflected different routes to generate energy from the fermented substrates in the gut.

Next, we examine more local environmental factors, such as diet and physical activity behaviors, and how these factors contribute to these relationships. Overall, diet-related microbiome associations [69, 70] have been more extensively studied; whereas, only a handful of studies have explored physical activity associations with the microbiota composition [71–75]. It is well described that the human microbiome is, in part, a reflection of the host’s diet, as exemplified by shifts in the infant microbiome following the introduction of solid food [76]. Specifically, this shift usually occurs between 18 and 36 months of age and was demonstrated by De Filippo et al. [77] who compared gut microbiota in healthy children, aged 1–6 years of age and either living in Italy or rural Burkina Faso. Interestingly, the average fiber content in the children from Burkina Faso was approximately 10–14 g/day versus 6–8 g/day in the Italian children and this difference in fiber and resistant starch intake was shown to result in Burkina Faso children to have greater levels of short-chain fatty acids in their fecal samples, than Italian children, and also reflected in different ratios of Firmicutes and Bacteriodetes between groups. Indeed other studies manipulating dietary resistant starch in the short term appear to result in greater changes in the gut microbiota compared to other dietary manipulations [78].

These results support other studies reporting that an individual’s dietary habits shape their microbial community and that this core community remains relatively stable during the host life span. Voreades et al. [70] reviewed several geographical studies comparing children and adults living in Malawi, and Venezuela, to participants living in the US [49], children living in India and the US [79] and finally South African and US African American adults [80] and collectively reported significantly different microbial communities, in part, as a result of differences in the macronutrient composition of the host’s diet [70]. Notably among the populations living outside of the US (Africans), where the diets are not as rich in protein and fat intake, there is an abundance of bacteria associated with resistant starches such as the Prevotella genus, as compared to US African Americans [80]. Schnorr et al. [69] reported that in traditional adult African hunter gathers living in Tanzania (Hadza), there was higher microbial richness and biodiversity compared to urban Italian adults, and their dietary habits were quite distinct (either hunter-gather related diet vs domestic farming, respectively). In another study examining urban vs rural individuals, microbial communities in stool samples of 50 adults from metropolitan and 46 adults from rural areas of Russia, using shotgun metagenomics, demonstrates that rural populations had a predominance of taxa associated with a ‘healthy gut’, which again mostly comprised differences in taxa associated with Firmicutes and Bacteroidetes [81]. The diets of the rural individuals were more in line with dietary data from populations living in developing countries compared to the diets of the urban samples, which were categorized by a reduced consumption of resistant starches, typically associated with the Western diet [81]. And finally, in a report examining the stool-associated microbiome of children from Europe and rural Africa, it was observed that significant correlations occurred with diet, whereby dietary fiber was a key driver of microbial differences between these geographically disparate cohorts [77].

Physical activity training has been shown to have significant, beneficial effects on the gut microbiota, by increasing the gut microbiota diversity and improving the ratio between certain bacterial genera [82]. One of the first studies to explore exercise-induced changes in microbial community structure found that in rats, voluntary training when compared to sedentary controls, significantly influenced the diversity of the microbiome [74], and also indicated that exercise enriched the cecum with butyrate-producing bacteria. In another report, Pertriz et al. [71] used obese and hypertensive rats and a treadmill running protocol, to expose rats to 4 weeks of exercise training program (5 days a week, for 30 min per day). Following the exercise training, both the obese and hypertensive rats showed increased microbiota diversity, associated with an increase in the relative abundance at the genus level in all rat models studied. In one of the few human studies, Clarke et al. [72] demonstrated that human individuals with higher levels of physical activity also have significantly greater microbial diversity in their stool; specifically the Firmicutes to Bacteroidetes ratio was greater in professional male athletes (mean BMI = 29 kg/m^2^) compared to two control groups, one with an overall higher BMI (mean 31 kg/m^2^) and one with an overall lower BMI (mean 23 kg/m^2^), to exclude the effects of body weight. Overall, these studies suggest exercise increases gut microbiota diversity.

The influence of exercise training on the gut microbiome has also been examined in combination with nutritional manipulations [73, 75]. Evans [73] explored the effects of 12 weeks of exercise training in combination with either a high or low fat diet, and the development of diet-induced obesity in mice, against sedentary controls. At the end of the study period, sedentary rats fed the high-fat diet gained considerably more body fat than the other groups, while the high-fat exercise rats experienced similar weight gains to the low-fat, sedentary rats. Importantly, however, exercise alone increased the ratio of Firmicutes to Bacteroidetes, irrespective of diet. Queipo-Ortuno et al. [75] further explored the combined effects of diet and physical activity on microbiota in rats assigned to different diet and exercise combinations, which included either diet restriction or ad libitum eating, and also unrestricted ad libitum exercise access or no exercise access. Significant effects were found most notably with rats exposed to exercise and energy restriction resulting in significant decreases in the quantity of Bacteroidetes and Firmicutes compared to the ad libitum groups. These studies provide the rationale for evaluating habitual physical activity levels when exploring the gut microbiota, across different geographical settings.

## Applying the epidemiologic transition model to understand the interaction of the obese gut-microbiota to geographical dependent factors

The aforementioned studies independently and collectively provide support for employing the epidemiologic model for studying the associations between the gut microbiota and the development of obesity, by allowing for the interplay between the individual and multiple (hierarchical) levels of causation or determinants (e.g. dietary habits, daily physical activity, socio-economic status, public health policy as well as access to health care). In fact, this model has been key to our understanding of obesity and also other chronic diseases in the modern world [39–41, 83–95]. However, the human gut microbiota and its implications for the obesity epidemic has just begun to be explored [49, 50, 77, 81]. Interestingly, and to the best of our knowledge, the microbiota has not been explored in relationship to the epidemiologic transition model. We, therefore, here focused this review on explaining our current understanding of the relationship between the gut microbiota and obesity, while considering some of the environmental factors of the host influencing the gut microbiota (of note, many other factors may contribute to the gut microbiota than discussed here). With exploring these variables through the epidemiologic transition model, we will be able to capture these interactions, and provide novel insight into the obesity epidemic.

## Conclusions

An epidemiologic model can be utilized to elucidate causal relationships between the gut microbiota and external environment independently and collectively, and this model will help us understand the role of the gut microbiota in the obesity epidemic. Notably, it is become well established that environmental factors influences the human gut microbiota, but it is still unclear exactly what environmental factors influence the gut microbiota in the development of the obese-phenotype. Unless we could control for all the environmental factors of human populations living in different areas of the world, which is unethical and impractical, the only way to fundamentally address this question outside of animal models is with large cross-sectional studies with sites across the globe. Hence we propose the development of large, globally-distributed, human microbiota studies to explicitly disentangle the interaction between environmental factors and the obese-phenotype associated gut microbiota.

## Abbreviations

WHO: World Health Organization
FTO: fat mass and obesity associated gene
HDI: human development index
US: United States
METS: Modeling the Epidemiologic Transition Study
